# Experimental comparative study of thrombogenicity of two differently luminal heparinized ePTFE vascular prosthetics

**DOI:** 10.1016/j.amsu.2018.09.037

**Published:** 2018-09-27

**Authors:** Mads Liisberg, Michael Stenger, Carsten Behr-Rasmussen, Jane Stubbe, Jes S. Lindholt

**Affiliations:** a Department of Cardiothoracic and Vascular Surgery, Odense University Hospital, Cardiovascular Centre of Excellence (CAVAC), Denmark; b Elitary Research Centre of Individualised Treatment of Arterial Diseases (CIMA), Odense University Hospital, Denmark; c Department of Vascular Surgery, Viborg Hospital, Denmark; d Department of Cardiovascular and Renal Research, Institute of Molecular Medicine, University of Southern Denmark, Denmark

## Abstract

**Introduction:**

; Heparin bonded grafts have proven to improve patency, at least transiently.

Two different heparin bonded expanded polytetrafluoroethylene (ePTFE) grafts produced by different technologies are currently available.

This pilot primary goal was to test these commonly used, but differently heparinized ePTFE grafts for differences in primary patency after a 6-months follow-up in a sheep model. Secondly, the aim was to establish a large animal model to enable future translational studies and further graft development.

**Method:**

; End-to-side bypass of the common carotid artery was performed bilaterally in sheep. Either a Gore^®^ Propaten heparinized graft or a Jotec^®^ Flowline Bipore heparinized graft was used, both 5 mm in diameter.

Following graft implantation, the sheep were kept on pasture for 6 months, with monthly duplex scans to determine patency. At termination, the grafts were duplex scanned a final time, with the animals sedated, and the grafts were removed for heparin activity analysis.

**Results:**

; 14 sheep were operated, 11 survived total follow-up time. At final follow-up, 4 patent Gore^®^ grafts, and 6 Jotec^®^ remained. Mean patency time was 106.7 ± 21.9(SD) days and 96.2 ± 25.9(SD) days for Gore^®^ and Jotec^®^, respectively. Log-rank test showed no significant difference at final follow-up after 6 months. Post mortem heparin analysis showed no significant difference in mean activity.

**Conclusion:**

; Based on patency data alone, no significant difference between these grafts were found. In accordance, heparin activity analysis showed no difference between the grafts. The model itself, proved easily implementable, and provides many possibilities for future studies, though some adjustments should be made to improve survival.

## Highlights

• First direct comparison of heparinized graft.
• No significant difference in patency or heparin activity concerning the two grafts.
• Animal model is easily implementable, and expandable to include numerous innovations.

## 1 Introduction

Revascularization below the knee, is typically done using autologous saphenous veins (ASV), as these are the conduit of choice. However, recent studies show, that ePTFE grafts bonded with heparin using the CARMEDA^®^ BioActive Surface (CBAS) process, have comparable patency to ASV [[1], [2], [3], [4], [5]]. Below the knee bypass/revascularization surgery is done to salvage limbs, but stenosis due to neointimal hyperplasia (NH) is the most common complication to revascularization surgery. This is especially true when utilizing artificial grafts [4].

A Swedish study from 1988 [6] showed heparin had an inhibitory effect on the formation of NH at the anastomosis site, in addition to the antithrombotic effect.

Ongoing development have improved the general patency of prosthetic grafts, although patency is still lacking compared to that of autologous vein grafts [[1], [2], [3], [4], [5],^7^]. The most recent development has been bonding heparin covalently to ePTFE grafts to ensure a sustained local heparin activity and potentially improve patency.

Additionally, risks such as acute hemorrhaging or heparin induced thrombocytopenia [8,9], associated with continuous heparin administration will not be an issue.

A Swedish company, Carmeda, achieved this and consequently patented their method, which was later purchased by Gore^®^. The first commercially available graft with CBAS bonded heparin, was released in 2004, around the same time a animal study showed a reduction in NH as well as improved patency when compared to crude grafts [10]. This was later confirmed in the Propaten-trial, a multicenter randomized clinical trial, including 11 Scandinavian centers and a total of 569 patients. This showed a significantly better overall patency of the heparinized grafts compared to ordinary PTFE grafts after 1-year follow-up [11].

Currently two name brands promote their grafts as being bonded with heparin. Gore^®^ warrants end-point bound heparin [12], anchoring the heparin-molecule with the bioactive-site reaching into the lumen of the graft, supposedly improving patency [11,13,14]. The Jotec^®^ graft is also marketed as coated with heparin, however Jotec^®^ does not specify how the heparin molecules are bound, therefore it could bound at random. This may reduce heparins efficiency as an anticoagulant, because the bio-availability may be reduced when compared to the Gore^®^ grafts. To our knowledge a study concerning this specific comparison was performed by Vermassen et al., in 2009, but so far, no results have been published [15].

This pilot study's objective was to investigate, the primary patency rates of these differently heparin bonded grafts, during a 6 months' follow-up period. Additionally, remaining heparin activity after 6 months would also be assessed, as well as the feasibility of the animal model itself.

## 2 Method and material

### 2.1 Design

This pilot study's design and experimental animal model was based on a previous study published by Pedersen et al. [10] using a paired design with bilateral implantation of grafts bypassing the common carotid arteries. This study however, directly compares two different Heparin bonded 5 mm grafts, as opposed to comparing a crude graft with a heparinized graft (both 6 mm in diameter) as Pedersen et al. did. Unlike Pedersen et al., we ligated the native common carotid artery approx. 1 cm distally from the proximal anastomosis, to create turbulent flow that would challenge the antithrombotic nature of the grafts.

The different grafts were alternately implanted on the right and left side by a fixed randomization key.

The study protocol was approved by the Danish animal experiments inspectorate.

### 2.2 Materials

The Gore^®^ grafts branded as Propaten^®^ grafts were 5 mm in diameter, and removable ring inforcing. They were made from expanded polytetrafluoroethylene, and had their rings removed to better match the Jotec^®^ grafts. Heparin is bound covalently to the graft surface, by a proprietary method allowing Gore^®^ to guarantee end-point bonding of the heparin molecules.

The Jotec^®^ grafts branded as FlowLine Bipore heparin^®^ were also 5 mm in diameter and had no ring reinforcement. The grafts were also made from expanded polytetrafluoroethylene. Jotec^®^ markets these grafts with innovative heparin coating, bound covalently, however makes no statement as to any specific binding of the heparin molecules.

### 2.3 Animals

Sheep were selected, because of their relatively stable body weight throughout a long follow-up period. Moreover, they usually recover rapidly following surgery with minimal care from animal technicians needed. This allows fast transfer from the stables to stay in pastures, which reduces costs and increases animal welfare.

We used female sheep (n = 14) wild type crossbreeds, but predominantly of the Texel race, which are usually more docile, thus easier to handle during duplex-scans without the need for sedation. Mean weight was 52 kg (range: 36–62 kg), mean age 4.2 years (range: 1–7 years).

### 2.4 Implantation of the grafts

All operations were done by the same two trained vascular surgeons, and a medical student assisting them, and during surgery an animal technician observed the vital parameters of the animal.

**Fig. 1:**
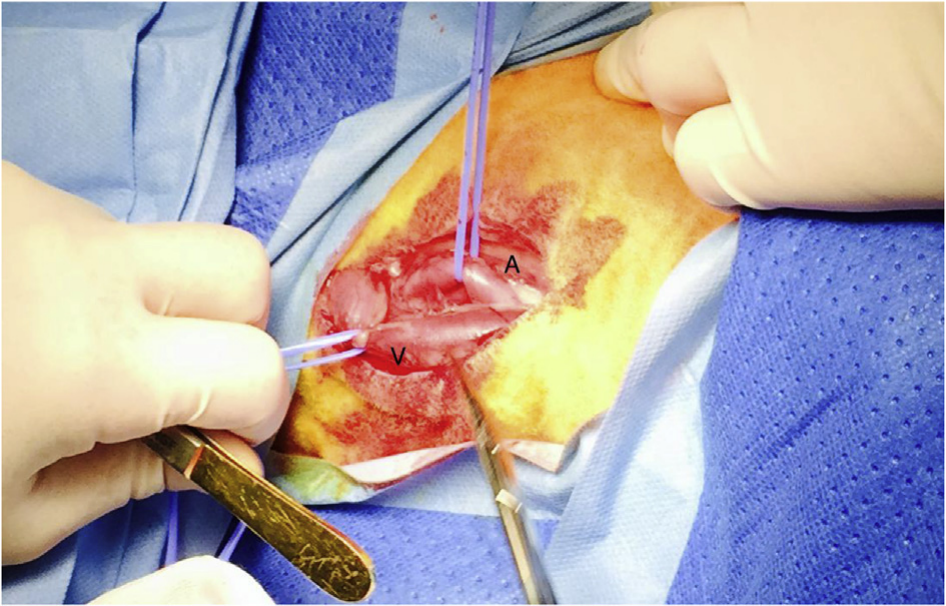
Preparation of the common carotid artery. A; Common Carotid Artery V; Jugular vein.

The common carotid artery was exposed for this bilaterally, using standard open surgical technique, exposing approximately 5–10 cm of the artery, depending on the muscular configuration of the sheep, to avoid damaging muscles, and the vagus nerve(Fig. 1). Both grafts were 5 mm in diameter, and the Gore^®^ graft which was ring-enforced had the rings removed to make it more comparable to the Jotec^®^ graft.

**Fig. 2:**
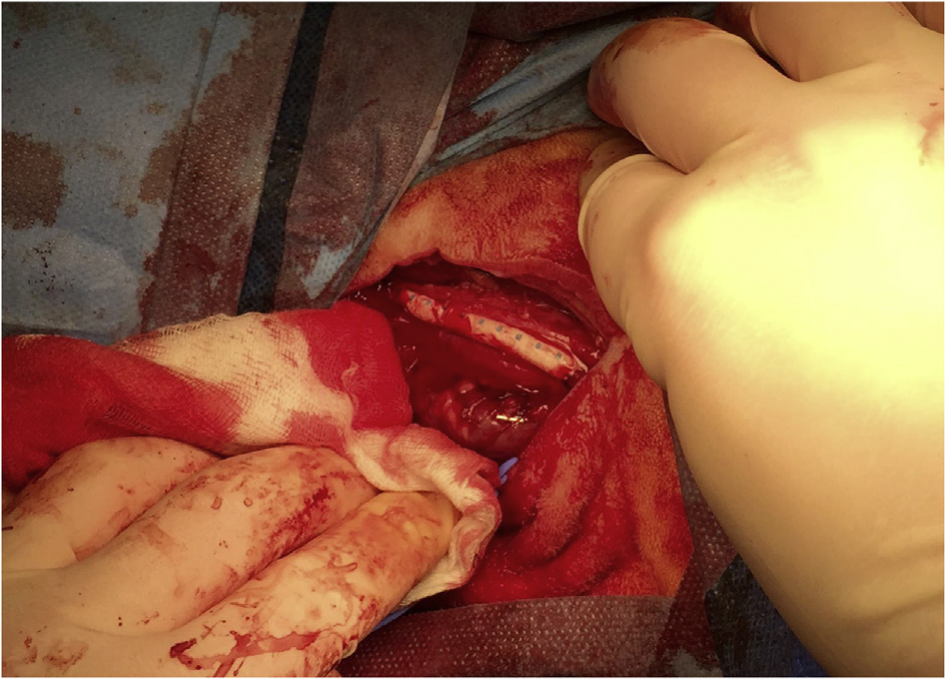
Final position of Gore^®^ graft, palpable pulse control of patency before wound closure.

Before clamping the common carotid artery, 5000 IU Heparin was administered, to prevent clotting, while suturing the anastomosis. Graft-ends were cut at a 45-degree angle and anastomosis were done in end-to-side fashion using 5–0 Prolene (Prolene^®^, B Braun, Melsungen, Germany) leaving the grafts approx. 6 cm in length. After confirming the patency of the anastomosis and the grafts themselves, with palpable pulse, the native artery was ligated using 2–0 prolene, approx. 1 cm distally from the proximal anastomosis(Fig. 2). The wound was closed in two layers, using self-absorbing 2-0 Vicryl (Vicryl^®^, Ethicon Inc., USA).

### 2.5 Follow-up

**Fig. 3:**
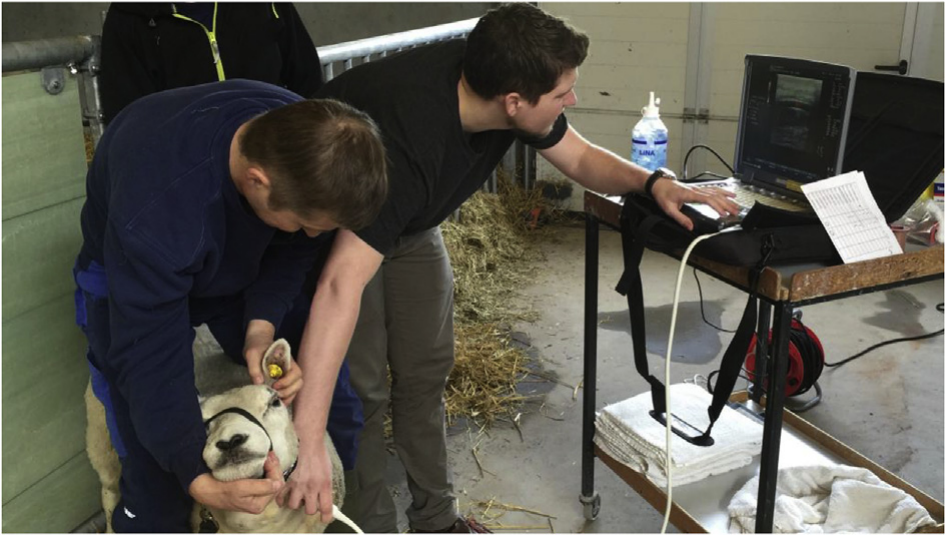
Duplex-scan of sheep.

All sheep were duplex scanned, with a portable GE Logiq E ultrasound device utilizing a linear probe (Fig. 3). Baseline scan was done two weeks following surgery, and every month following that, for a complete follow up of 6 months all duplex scans were carried out by the same operator. This protocol was continued even after an occlusion was detected.

Additionally, the sheep were also be duplex scanned during sedation right before sacrificial. Immediately before the sheep were sacrificed, 5000 IE heparin was administered to prevent blood clots in the prosthesis, this was done to enable easier in-vitro analysis.

The remaining heparin activity from the removed grafts, after 6 months was analyzed at Carmeda AB laboratories in Stockholm, Sweden.

### 2.6 Statistical analysis

Sample size was derived from power calculations, with 0.8 power at a 0.05 significance level and a 0.2 correlation coefficient, which suggested a sample size of 11, but to account for eventual losses 14 sheep were operated.

Patency for each graft was recorded, in days, and difference between grafts was tested using log-rank analysis. Post hoc analysis after 3 months were performed. The difference in patency was further analyzed by paired *t*-test of the differences in patency's between the individual paired grafts.

Additionally, survival analysis was graphed as a Kaplan-Meier plot. Stata/IC version 14 (Statacorp) were used for all analyses and illustrations.

The heparin activity was expressed as quantitative results, which represented the amount of active heparin at removal at the end of follow up. The differences between the individual implanted grafts were compared by paired *t*-test.

## 3 Results

### 3.1 Surgical outcome

14 Sheep were operated, one had to be put down 3 days following the operation, due to post-operative complications. Another was euthanized after 9 days due to suspected aspiration pneumonia. Lastly one sheep had a wound defect, resulting in a prosthesis infection, which had to be removed. The same animal was put down at the first monthly follow-up, possibly due to paralytic bowels syndrome. The remaining animals did not suffer any complications and stayed on pasture for the entire follow-up period.

### 3.2 General patency

After 6 months follow up, n = 4 (36.4%) Gore^®^ and n = 6 (54.5%) Jotec^®^ grafts were still patent (Table 1). It should be noted, that the sheep, which was euthanized due to a wound defect, still had a functioning Gore^®^ graft.

**Table 1:** Number of patent grafts at baseline duplex scan – and at 6 month endpoint.

	Baseline	Endpoint
Gore n = 11	9 (81.8%)	4 (36.4%)
Jotec n = 11	7 (63.6%)	6 (54.5%)

### 3.3 Patency in days

Mean patency for the Gore^®^ grafts were 116.4 ± 21.6 (SD) days compared to Jotec^®^ 104.9 ± 26.7(SD) days. Paired t–test analysis of the difference between the pairs of grafts showed no significant difference (p = 0.74).

Heparin activity was recorded as picomol/cm^2^ for each graft. Mean heparin activity in Gore^®^ grafts was 16.6 pmol/cm^2^ ± 2.3 (SD), and 15.6 pmol/cm^2^ ± 4.3 (SD) for the Jotec^®^. Paired *t*-test showed no difference between grafts, with a mean difference of 1.00 (95% CI -2.64; 4.64), p = 0.554.

The Kaplan-Meier plot (Fig. 6), shows that the Gore^®^ grafts did not occlude as rapidly as the Jotec^®^ grafts with 6 patent grafts of each make after three months. However, after this initial period an increasing number of Gore^®^ graft failures were observed, and at six months’ final follow up, the initially observed difference between the grafts had diminished (p = 0.88 by log-rank test) with four remaing patent Gore^®^ grafts and six Jotec^®^ grafts.

Interestingly, post-hoc analysis, showed that the Gore^®^ grafts, had significantly better patency than the Jotec^®^ grafts until the 3rd month, were most of the occlusions occurred in both groups (p = 0.027 by log-rank test).

### 3.4 Visibility

**Fig. 4:**
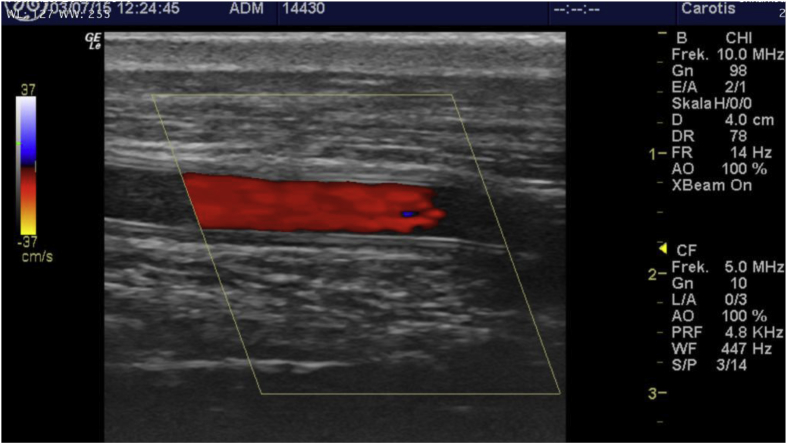
Open graft, no turbulent flow or signs of thrombosis.

**Fig. 5:**
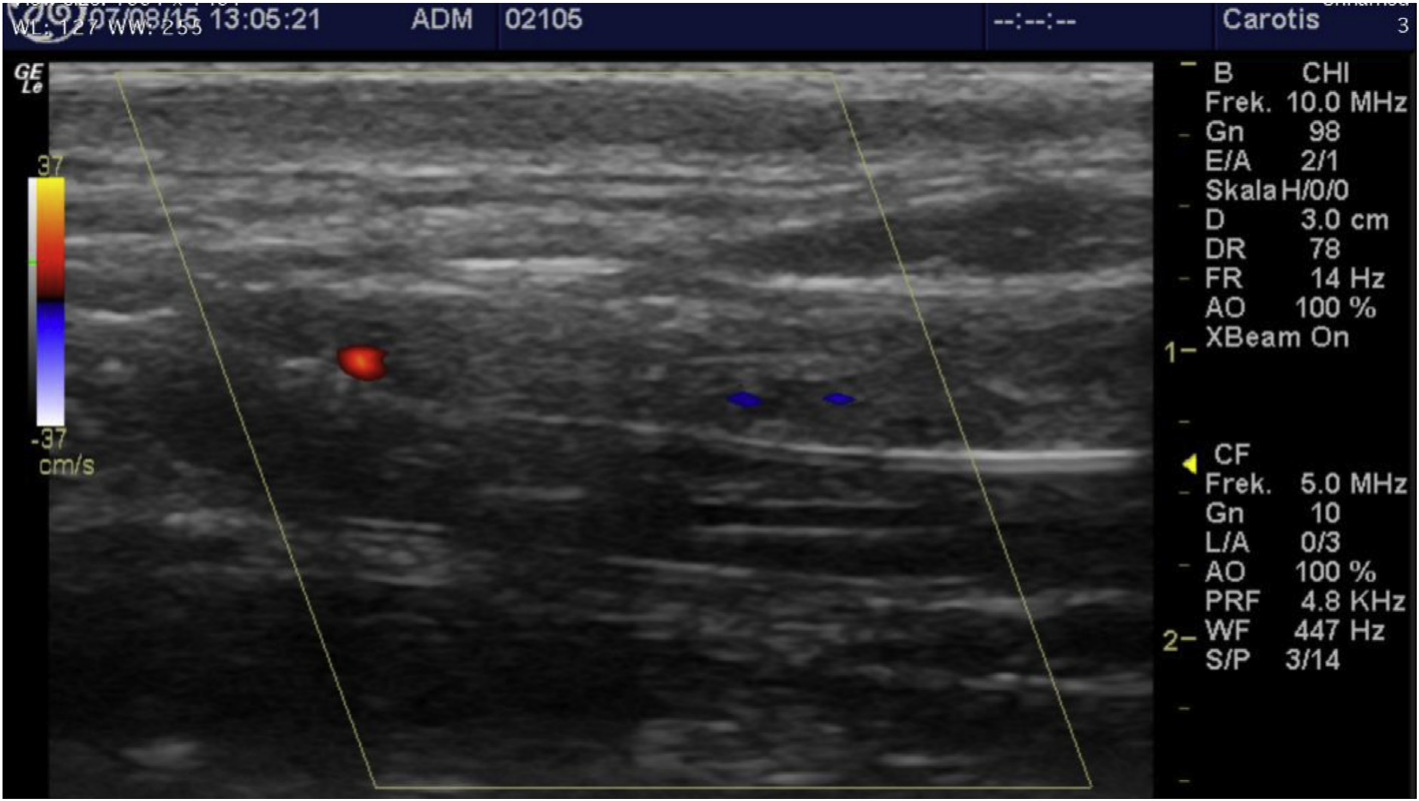
Occluded graft, no color signal. Mural thrombosis is clearly visible. (For interpretation of the references to color in this figure legend, the reader is referred to the Web version of this article.)

Concerning ultrasonic visibility of the implanted grafts, it is notable that duplex scans were carried out with the animals un-sedated. This was done to reduce the stress on the animals, sometimes requiring operator patience. Depending on the muscular configuration of the sheep, and how the grafts themselves behaved in-vivo, visibility was remarkably good, with clear duplex signal (Fig. 4), and in the case of occlusion, even visible thrombosis (Fig. 5). The animals were sedated before sacrifice, and only 2 duplex-scans differed from the previous scanning results. Concordantly if a graft was patent at the time of sacrifice and previous scans had deemed it occluded, it was recorded as a patent graft throughout the study.

## 4 Discussion

Heparin bonded grafts have proven to improve patency, at least transiently [16].

Differences in heparin bonding to the grafts may influence the outcome.

This pilot study demonstrates, as the first comparative study, no immediate significant difference, in primary patency between the tested grafts at 6 months’ follow-up. However a trend showing a potential short-term effect is found. Due to the high numbers and long observation time needed in clinical trials, examining different grafts patency is often done retrospectively in registry studies [1,7], which make a controlled experimental study like this desirable. This study also paves the way for future animal studies to investigate novel advancements into artificial grafts, as the animal model itself shows great potential.

**Fig. 6:**
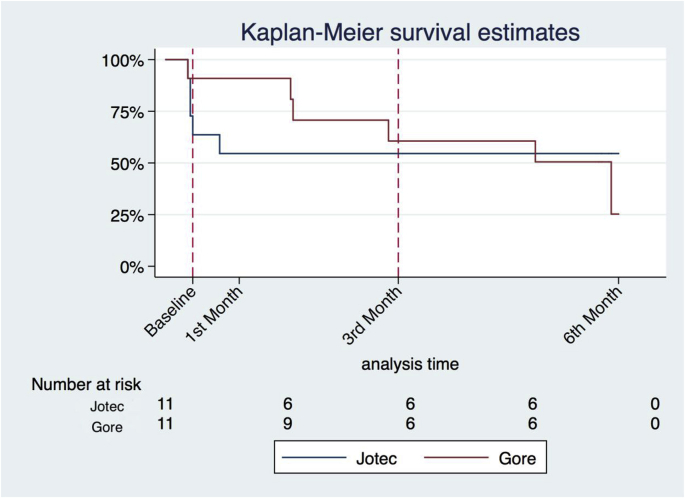
Kaplan-Meier survival plot of Gore^®^ and Jotec^®^ Grafts. Log-rank p = 0.88 at end-point.

Concerning our findings regarding patency, the Kaplan-Meier plot (Fig. 6) suggest no significant (p = 0.88) difference between the grafts after 6-months follow up.

Nevertheless, there seems to be a trend indicating that initial patency is better in the Gore^®^ grafts, with n = 2 (18.2%) failures at the time of first duplex, compared to n = 4 (36.4%) Jotec^®^. As previously shown the crucial point remains at 3 months [10], where many occlusions occur. However, at this point log-rank test still shows a significant difference between the grafts (p = 0.027). This could be due to the sustained heparin activity in the Gore^®^ grafts, which might protect the grafts from acute occlusion within the first three months. Additionally, if our hypothesis is right, it also explains why the Jotec^®^ grafts occlude earlier on. However, this remains speculative as it was not planned initially and would require additional heparin analysis, but it does show an interesting trend.

In comparison with other animal studies investigating heparin bonded grafts, the occlusion rate of this series is higher than expected [13,17,18]. This might be partly explained by a smaller diameter of the grafts used, since comparable studies used 6 mm grafts opposed to 5 mm in our study, resulting in a 30% decrease in lumen area, increasing the strain on the grafts.

Looking at the removed grafts reported heparin activity, no difference was found. This corresponds with our survival analysis. Retrospectively it could have been interesting to perform the heparin activity analysis both prior to implantation and after explanation.

### 4.1 Methodological considerations

Our intension was to challenge the bonded heparin and patency of grafts as much as possible by choosing 5 mm grafts and furthermore, creating turbulent flow by ligation of the native common carotid artery approximately 1 cm distally from the proximal anastomosis. These modifications of the model may have overly encouraged thrombogenicity, compared to previous reports [10]^,^ [13,17,18]. This might also contribute to the relative high number of occlusions, which is not in line with previous retrospective studies with a 70–80% patency rate in diseased patients [11,14,19]and in contrast to the reported patency by Pedersen et al.

Additionally, patency studies done in baboons, canines, and sheep all show better patency in grafts with CBAS immobilized heparin [10]^,^ [13,17,18], than what this study reports.

If a larger diameter graft had been used, there might have been a clearer difference between the grafts, than in this current setup. The strain on the grafts in our series, might censor the actual difference between the grafts.

It could be argued that antiplatelet therapy, which ought always to be administered to patients with known atherosclerotic disease, would improve the patency of these grafts as shown by an arteriovenous-graft model in porcine [17]. However, the interest of this study is purely in the anti-thrombotic abilities of the grafts themselves. Which is why antiplatelet therapy, was not included in this current series.

### 4.2 Study limitations

In our series, there were some early censoring of the grafts, following the complications mentioned above. These could have been due to surgery, although all surgical procedures were performed proficient, and up to clinical standards. One graft however, did get infected most likely because of a wound defect, and not because surgical aseptic failure.

Considering that three out of all 14 (21.4%) sheep did not live through the length of the study, this is of course a disadvantage to the series, but on the other hand, the required sample size was achieved.

Another limitation of this series, and potentially in any series using sheep, is that their atherosclerotic status, is unknown, if there is any. This could have potential implications when using data derived from sheep studies, when treating patients.

It is still too early to reach a conclusion concerning the differences in primary patency between these two types of heparinized grafts. Mainly because our model might have challenged the grafts to a clinically irrelevant level. But this could probably be avoided using a larger diameter graft in a future series. It could also be argued that the grafts could be interpositioned with end-to-end anastomosis instead of end-to-side, to reduce the strain on the grafts, when testing the antithrombotic capabilities. But this would not reproduce real-world conditions for most below knee bypasses.

### 4.3 Clinical implications

This study, has continued the foundation of earlier animal studies, to create a large model that allows a direct comparison of grafts.

This large animal model makes a direct comparison a real possibility. We are now able to compare the effects of these modern grafts in-vivo, which provides us with an extended evidence base to choose the best treatment available for patients.

Regarding the patency results, more research is still required. This could be done using a graft size which is more commonly used in revascularization surgery, i.e. a 6 mm graft. This might reduce the strain on the grafts, and therefore provide a more accurate result. A future study could include a 3-month end-point, were grafts would be removed for heparin analysis, since it seems that most occlusions occur around this point in time.

This study model shows promise to enable future studies to be conducted investigating the future development of artificial vascular grafts. It could even be adapted to feature an infection model, that could test grafts for their ability to fight of infections, or timed drug release.

## Disclosures

We reached out to both companies, however only Gore^®^ responded and chose to provide the utilized grafts. The Jotec^®^ grafts were bought through the vascular surgery department at Odense University Hospital. Additionally, the Carmeda^®^ labs performed the heparin analysis of the removed graft material.

## Ethical approval

The study protocol was approved by the Danish animal experiments inspectorate.

## Sources of funding

Funding was obtained at University Hospital Odense Grant ID 15-A915 and through Aase & Ejnar Danielsens fond Grant ID 10-001454.

## Author contribution

Mads Liisberg/Jes Lindholt – Conceptualisation, planning and funding.

Mads Liisberg/Michael Stenger/Carsten Behr-Rasmussen – Surgery, analysis and follow-up.

Mads Liisberg – Writing.

Jane Stubbe/Jes Lindholt/Michael Stenger/Carsten Behr-Rasmussen – editing.

## Conflicts of interest

Gore^®^ provided grafts for the study – we reached out to Jotec but they did not reply after several advances.

Heparin analyses was conducted at Carmeda labs.

## Research registration number

Not relevant.

## Guarantor

Mads Liisberg, M.D.

## Provenance and peer review

Not commissioned, externally peer reviewed.
